# Supplementary material for: Melatonin Alleviates Lipopolysaccharide-Induced Endometritis by Inhibiting the Activation of NLRP3 Inflammasome through Autophagy
Source: Animals (Basel). 2023 Jul 28;13(15):2449. doi: 10.3390/ani13152449 (PMC10417527; doi:10.3390/ani13152449)

Supplementary Figure S1. Western blots raw data showing all bands with molecular weight markers for corresponding figures.

Figure 2 Melatonin suppresses the NLRP3 inflammasome activation in LPS-stimulated BEND cells

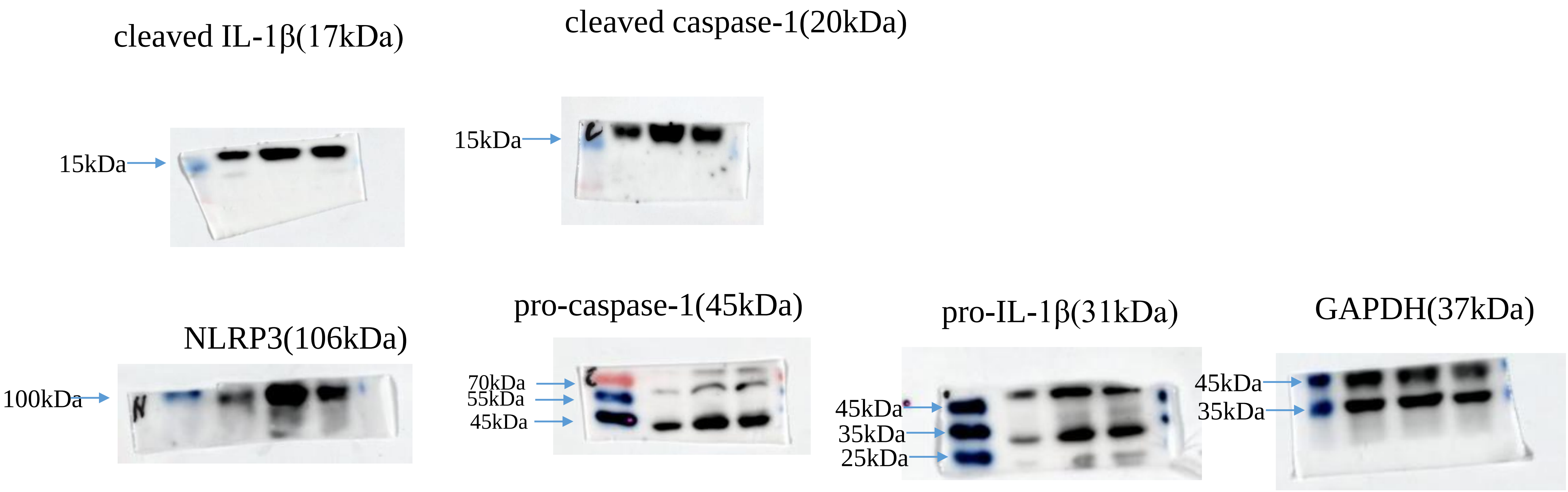

Figure 3 melatonin promoting autophagy in LPS-induced BEND cells

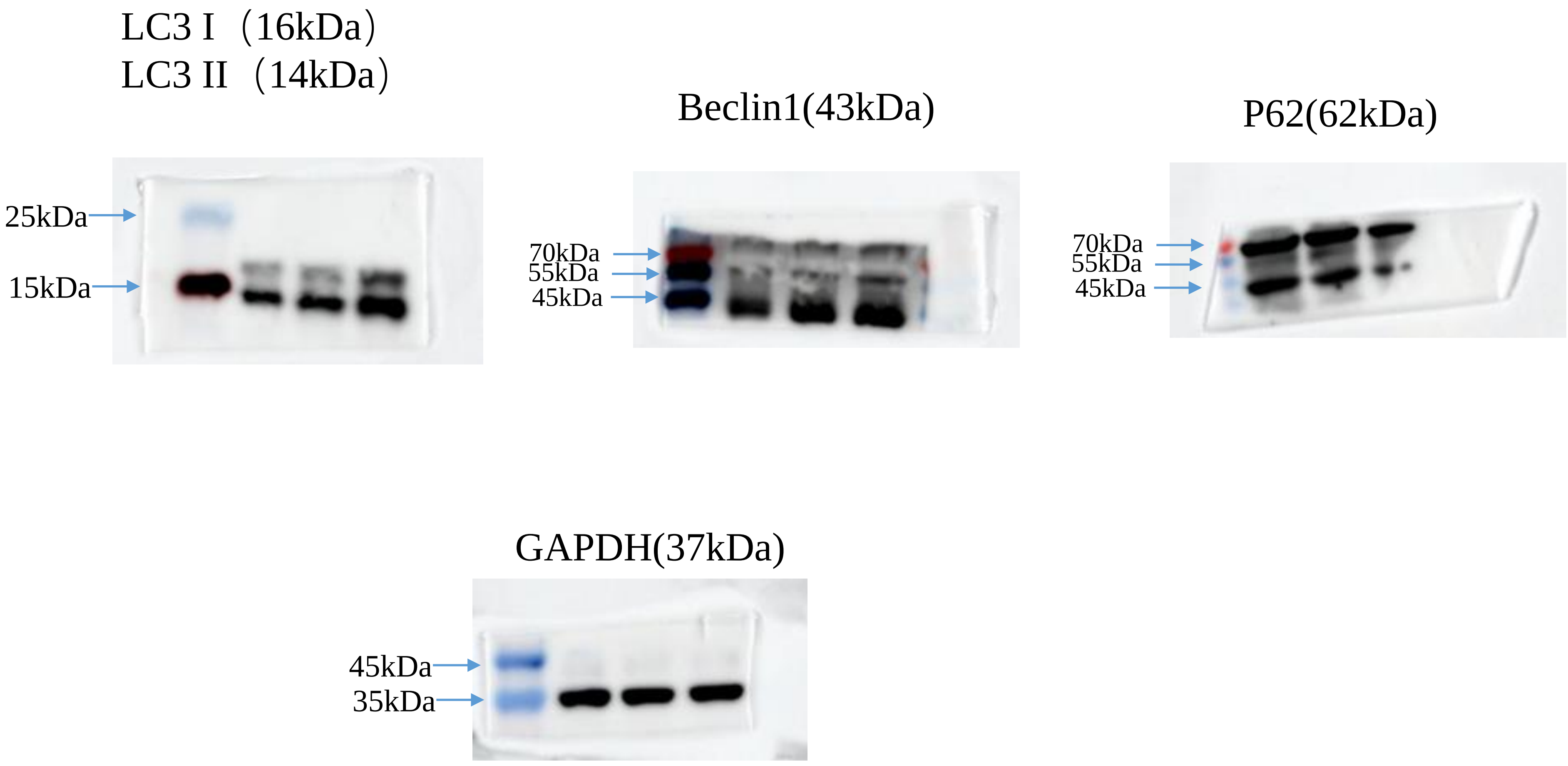

Figure 4 Melatonin promotes mitophagy and alleviates mitochondrial dysfunction in LPS-induced BEND cells

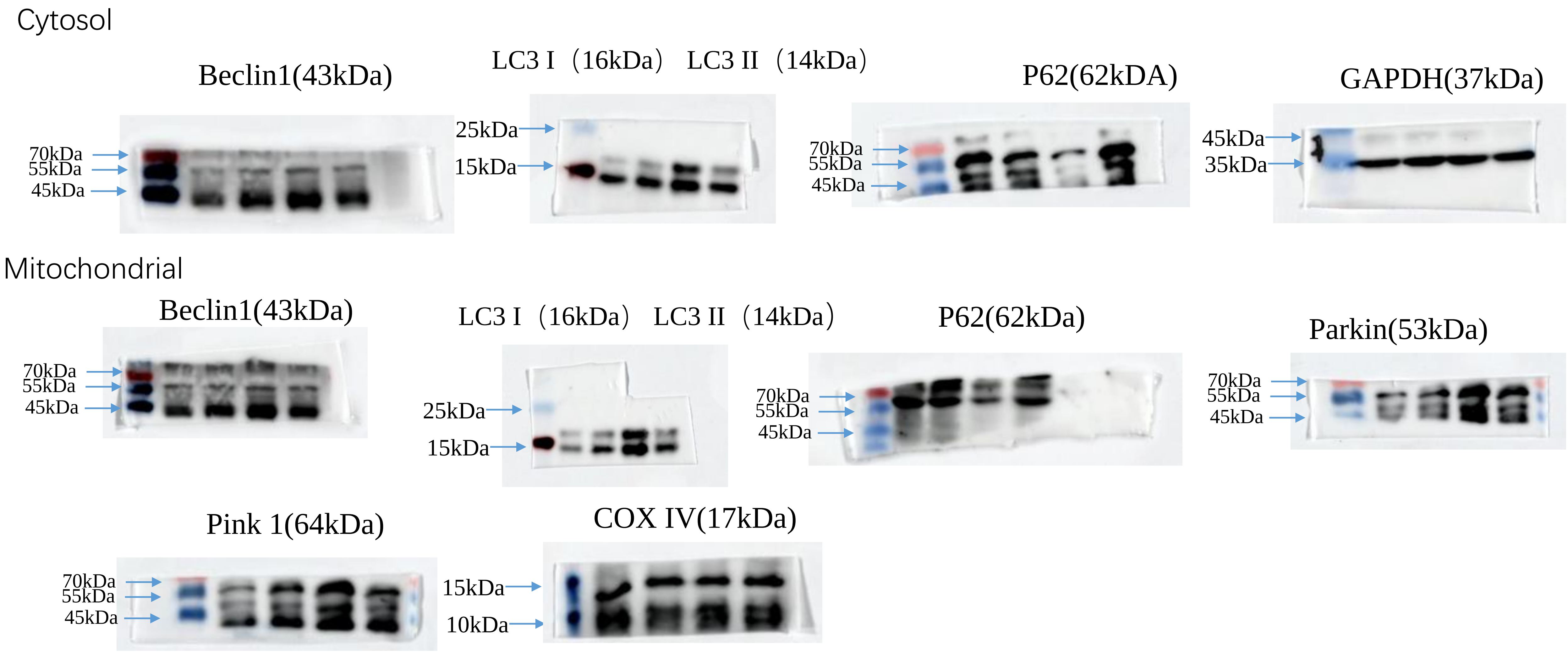

Figure 5 3-MA reverses the inhibitory effect of melatonin on inflammation and mitochondrial dysfunction in LPS-induced BEND cells

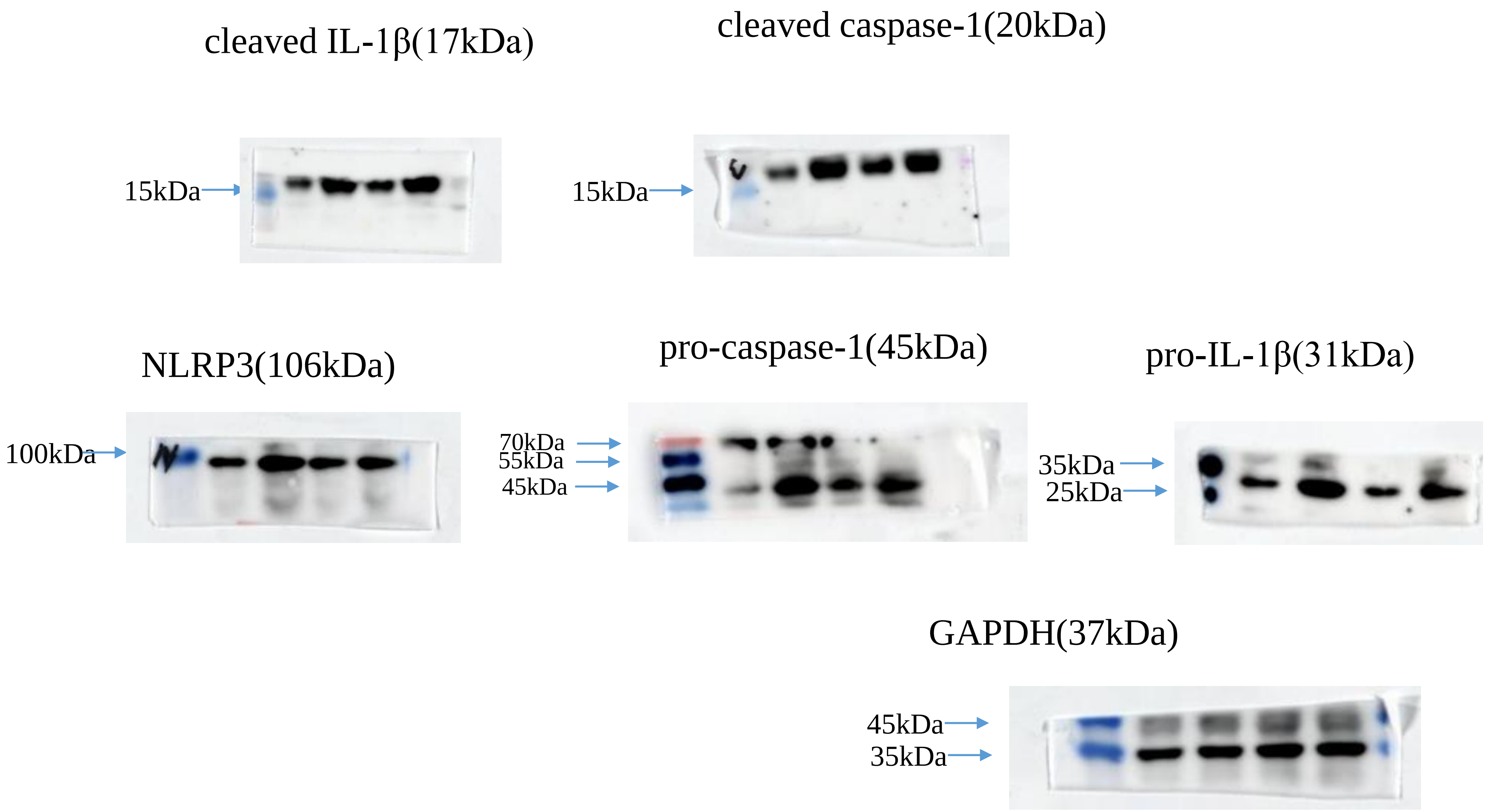

Supplement: Supplementary file 1 [file animals-13-02449-s001.zip › animals-2484589-supplementary.pdf]
